# MicroRNA‐138 Inhibits Osteogenic Differentiation and Mineralization of Human Dedifferentiated Chondrocytes by Regulating RhoC and the Actin Cytoskeleton

**DOI:** 10.1002/jbm4.10071

**Published:** 2018-07-18

**Authors:** Hongjun Zheng, David Ramnaraign, Britta A Anderson, Eric Tycksen, Ryan Nunley, Audrey McAlinden

**Affiliations:** ^1^ Department of Orthopaedic Surgery Washington University School of Medicine St Louis MO USA; ^2^ St Louis University School of Medicine St Louis MO USA; ^3^ Genome Technology Access Center Washington University School of Medicine St Louis MO USA; ^4^ Department of Cell Biology Washington University School of Medicine St Louis MO USA

**Keywords:** MICRO‐RNA, MIR‐138, DEDIFFERENTIATED CHONDROCYTES, OSTEOGENESIS, MINERALIZATION, RHOC, ACTIN CYTOSKELETON

## Abstract

MicroRNAs (miRNAs) are known to play critical roles in many cellular processes including those regulating skeletal development and homeostasis. A previous study from our group identified differentially expressed miRNAs in the developing human growth plate. Among those more highly expressed in hypertrophic chondrocytes compared to progenitor chondrocytes was miR‐138, therefore suggesting a possible role for this miRNA in regulating chondrogenesis and/or endochondral ossification. The goal of this study was to determine the function of miR‐ 138 in regulating osteogenesis by using human osteoarthritic dedifferentiated chondrocytes (DDCs) as source of inducible cells. We show that over‐expression of miR‐138 inhibited osteogenic differentiation of DDCs in vitro. Moreover, cell shape was altered and cell proliferation and possibly migration was also suppressed by miR‐138. Given alterations in cell shape, closer analysis revealed that F‐actin polymerization was also inhibited by miR‐138. Computational approaches showed that the small GTPase, RhoC, is a potential miR‐138 target gene. We pursued RhoC further given its function in regulating cell proliferation and migration in cancer cells. Indeed, miR‐138 over‐expression in DDCs resulted in decreased RhoC protein levels. A series of rescue experiments showed that RhoC over‐expression could attenuate the inhibitory actions of miR‐138 on DDC proliferation, F‐actin polymerization and osteogenic differentiation. Bone formation was also found to be enhanced within human demineralized bone scaffolds seeded with DDCs expressing both miR‐138 and RhoC. In conclusion, we have discovered a new mechanism in DDCs whereby miR‐138 functions to suppress RhoC which subsequently inhibits proliferation, F‐actin polymerization and osteogenic differentiation. To date, there are no published reports on the importance of RhoC in regulating osteogenesis. This opens up new avenues of research involving miR‐138 and RhoC pathways to better understand mechanisms regulating bone formation in addition to the potential use of DDCs as a cell source for bone tissue engineering. © 2018 The Authors. *JBMR Plus* is published by Wiley Periodicals, Inc. on behalf of the American Society for Bone and Mineral Research.

## Introduction

Micro‐RNAs (miRNAs) are small noncoding RNAs that function at the posttranscriptional level to suppress translation and/or induce degradation of target mRNAs following binding to specific sites within their 3'UTRs.^1^ In a given cell type, tens to hundreds of genes may be targeted and downregulated by a single miRNA resulting in modulation of multiple cellular pathways.^2^ As such, miRNAs are recognized as important regulators of a diverse range of physiological processes including cell proliferation, differentiation, migration, and apoptosis. Dysregulation of miRNAs has also been identified in a number of pathological states including cancers, cardiac disease, hepatitis C, and various musculoskeletal conditions including osteoarthritis and osteoporosis.^3^, ^4^, ^5^, ^6^, ^7^

With respect to bone biology and disease, numerous studies have reported functional roles for many miRNAs in regulating osteogenesis and bone cell homeostasis.^7^, ^8^, ^9^ Among some well‐studied bone‐related miRNAs, miR‐214 has been identified as an inhibitor of osteogenic differentiation, and delivery of miR‐214 antagomirs in vivo was found to attenuate bone loss in mice.^10^, ^11^, ^12^, ^13^, ^14^ Mechanistically, miR‐214 was shown to inhibit *ATF4* to inhibit osteoblastogenesis,^10^ whereas it was also shown to induce osteoclastogenesis by targeting *Pten*.^15^ Another miRNA, miR‐138, has also been shown to function as an inhibitor of mesenchymal stem/stromal cell (MSC) osteogenesis via suppression of the focal adhesion kinase (FAK) signaling pathway; ex vivo approaches indicate that miR‐138 antagomirs could enhance bone formation.^16^, ^17^ The primary mir‐138 sequence is located at two regions in the human genome (chromosomes 3 and 16) resulting in production of two mature miRNAs: miR‐138‐1 and miR‐138‐2. The functional 5p strand of this miRNA is conserved between humans and mice. In the cancer field, miR‐138 functions as a tumor suppressor and has been shown to regulate a number of processes including cell proliferation, apoptosis, migration, and invasion.^18^ In addition, miR‐138 has also been reported to induce pluripotency via regulation of p53.^19^ A previous study from our laboratory has identified differentially expressed miRNAs within the developing human tibia and femur.^20^ Among these, expression of miR‐138 was found to be more highly expressed in hypertrophic chondrocytes compared with chondro‐progenitor cells. This suggests a potential role for miR‐138 in regulating chondrogenesis and/or endochondral ossification.

Although mesenchymal stem/stromal cells (MSCs) are a commonly used progenitor cell source to study cell differentiation in the chondrogenic and osteogenic lineages,^21^ human primary articular chondrocytes that have undergone multiple passages (P4 or higher) are another source of cells with multipotent function. These dedifferentiated chondrocytes (DDCs) acquire a fibroblastic phenotype and express higher levels of type I collagen (*COL1A1*) relative to the chondrocyte marker, type II collagen (*COL2A1*). Studies have shown that DDCs can be redifferentiated back toward the chondrogenic lineage^22^, ^23^, ^24^, ^25^; of clinical relevance, the ability of these cells to induce cartilage repair or formation has been demonstrated in animal models as well as 3D‐engineered microfibers.^26^, ^27^, ^28^ Interestingly, differentiation of DDCs toward the osteogenic and adipogenic lineages has also been demonstrated in vitro,^29^ yet the mechanisms regulating these differentiation programs in DDCs remain unexplored.

In this study, we have utilized DDCs isolated from human osteoarthritic articular cartilage to determine the function of miR‐138 in regulating osteogenic differentiation of these cells. We found that miR‐138 inhibits osteogenesis and matrix mineralization and noted that cell proliferation, cell shape, and cell migration were also affected. Analysis of potential miR‐138 target genes led us to pursue one specific target, RhoC, because this small‐molecular‐weight GTPase has been implicated in altering cell shape via regulation of the actin cytoskeleton, in addition to regulating processes controlling cell proliferation, migration, and invasion in other systems.^30^, ^31^, ^32^, ^33^, ^34^ Here, we have discovered a new mechanism in DDCs, whereby miR‐138 functions to suppress RhoC expression, which in turn inhibits F‐actin polymerization and the ability of these cells to proliferate, possibly migrate, and differentiate toward the osteogenic lineage. Our additional findings that knockdown of RhoC alone can inhibit osteogenesis provides further evidence to support a functional role for RhoC in regulating the osteogenic process. This opens up new avenues of research involving miR‐138 and RhoC signaling pathways as a means to better understand mechanisms regulating bone formation, in addition to the potential use of DDCs as a cell source for bone tissue engineering.

## Materials and Methods

### Isolation and dedifferentiation of human primary chondrocytes

Human articular cartilage tissue was obtained from osteoarthritis (OA) patients following total knee replacement surgery. Utilization of tissue was approved by the Washington University Human Research Protection Office (IRB ID# 201104119). Articular cartilage was removed from the joint surface, diced into small pieces (approximately 2 mm^3^), and digested in growth medium (DMEM/F12; 10% FBS) containing 0.025% collagenase and 0.025% pronase in cell‐spinner flasks overnight at 37°C. The resulting cell suspension was washed in Hank's balanced salt solution and processed through a 70‐μm sterile filter. Primary chondrocytes were seeded in T75‐cell‐culture flasks (1 × 10^6^ cells/flask) and cultured in growth medium until 90% confluency. Cells were passaged at least 4 times and dedifferentiation was confirmed by assessing the ratio of *COL1A1*:*COL2A1* expression by qPCR.

### Lentiviral production and transduction

Human genomic pri‐miR‐138 and approximately 150 nucleotides upstream and downstream of the pri‐miRNA sequence were amplified by PCR (see Table 1 for primer sequences). The miR‐138 amplicon was inserted into the pLemiR backbone (Addgene, Cambridge, MA, USA)^35^ using Gibson Assembly Master Mix (New England Biolabs, Ipswich, MA, USA). The integrity of the resulting clones was confirmed by Sanger sequencing. pLemiR lentiviruses were used to overexpress the pri‐miR‐138 or a nonsilencing (NS) control RNA.^35^ Lentiviral stocks were prepared as previously described,^36^ and titered using the Lenti‐X p24 rapid titer ELISA (Clontech Laboratories, Palo Alto, CA, USA). Dedifferentiated chondrocytes (DDCs) were seeded at 2 × 10^5^ cells/well in 12‐well plates and transduced for 24 hours with pLemiR lentiviruses expressing the NS control (LV‐NS) or miR‐138 (LV‐138) at a multiplicity of infection (MOI) of 20 using growth medium containing 100 μg/mL protamine sulfate. Fresh growth medium was applied 24 hours after transduction. Transduced DDCs were cultured in growth medium for an additional 48 hours prior to the addition of osteogenic induction medium.

**Table 1:** Primer sequences and Life Technologies miRNA assay IDs used for vector cloning and quantitative PCR

Amplicon	Forward primer (5’ – 3’)	Reverse primer (5’ – 3’)	NCBI Reference
miR‐138 Genomic	CAAACTGGGGCACAGATAACTGGGG AAGGCAGTGAAAT	GGGAGAGGGGCGGAATTTGCGGGGG ATAAACAGCAGCC	NR_029700.1
miR‐138	Life Technologies TaqMan miRNA assay ID 002284		NR_029700.1
miR‐21	Life Technologies TaqMan miRNA assay ID 000397		NR_029493.1
RNU44	Life Technologies TaqMan miRNA assay ID 001094		NR_002750
*PPIA*	TCCTGGCATCTTGTCCATG	CCATCCAACCACTCAGTCTTG	NM_021130.4
*COL2A1*	GGCAATAGCAGGTTCACGTACA	CGATAACAGTCTTGCCCCACTT	NM_001844.4
*COL1A1*	TTCCCCCAGCCACAAAGAGTC	CGTCATCGCACAACACCT	NM_000088.3
*RUNX2*	CATCACTGTCCTTTGGGAGTAG	ATGTCAAAGGCTGTCTGTAGG	NM_001024630.3
*OSX*	CCACCTACCCATCTGACTTTG	CCTTCTAGCTGCCCACTATTT	AF477981.1
*BSP*	TGCTACAACACTGGGCTATGGA	CTTCTTGGGAAGCTGGATTGC	NM_004967.3
*OPN*	CATATGATGGCCGAGGTGATAG	AGGTGATGTCCTCGTCTGTA	NM_001040058.1
*OCN*	AAATAGCCCTGGCAGATTCC	CAGCCTCCAGCACTGTTTAT	NM_199173.5
*ALP*	GAAGTGGGAGTGCTTGTATCT	GAGGCAGTGGAGACAGATTTAG	NM_000478.5
*MMP13*	CTTGACCACTCCAAGGACCC	CCTGGACCATAGAGAGACTGGA	NM_002427.3
*RhoC*	GTCATCCTCATGTGCTTCTC	CTTGCCTCAGGTCCTTCTTATT	NM_175744.4

Life Technologies = Life Technologies Inc, Grand Island, NY, USA; NCBI = National Center for Biotechnology Information.

### Osteogenic differentiation of DDCs

Nontransduced or lentiviral‐transduced DDCs were seeded in 12‐well plates at a density of 2 × 10^5^ cells/well in osteogenic differentiation medium (αMEM containing 10% FBS, 2mM L‐glutamine, 100 U/mL penicillin, 100 µg/mL streptomycin, 10mM β‐glycerol phosphate, 50 µM ascorbic acid, 10nM dexamethasone) for up to 14 days with fresh medium changes every 3 days. To examine matrix mineralization at day 14, DDCs were fixed in 4% paraformaldehyde and stained for 30 min with 1% Alizarin Red ethanol solution. Alkaline phosphatase activity was monitored in day 14 paraformaldehyde‐fixed DDC cultures following treatment with 1‐step 4‐nitro blue tetrazolium/5‐bromo‐4‐chloro‐3‐indolyl phosphate substrate solution (Thermo Fisher Scientific, Waltham, MA, USA) for 15 min at 37°C in a dark humidified chamber. Bone‐specific matrix hydroxyapatite was detected in day 14 cultures using the OsteoImage Mineralization Assay Kit (Lonza Group, Basel, Switzerland) following the manufacturer's protocol. Fluorescence microscopy was carried out for semiquantitative analysis of hydroxyapatite levels. Data were quantified on a fluorescent plate reader set to the appropriate excitation/emission wavelengths (492 nm/520 nm).

### RNA isolation and qPCR

Total RNA was extracted from cells using the Total RNA Purification Kit (Norgen Biotek Corp, Thorold, ON, Canada), which isolates both miRNAs and mRNAs. miRNAs were reverse‐transcribed and quantified with the appropriate TaqMan primer/probe sets (Life Technologies Inc, Grand Island, NY, USA; Table 1), using the TaqMan microRNA reverse transcription kit (Life Technologies) and TaqMan master mix with no UNG (Life Technologies). To determine if levels of miR‐138 following lentiviral transduction were within physiological range, we compared copy numbers of miR‐138 and miR‐21 (a known highly expressed miRNA) in DDCs following transduction with LV‐NS or LV‐138. Synthetic miRNAs corresponding to the 5p strand of mature miR‐138 or miR‐21 were generated (IDT) and known copy numbers (ranging from 100 million to 10 copies) were reverse‐transcribed and amplified by qPCR. Standard curves plots showing miRNA copy number and corresponding CT values were generated and these were used to calculate copy numbers of either miR‐21 or miR‐138 in DDCs following transduction with LV‐NS or LV‐138. mRNAs were reverse‐transcribed using Superscript RT II (Life Technologies), and quantitative PCR was performed using PowerUp SYBR master mix (Life Technologies). PCR primer sequences are shown in Table 1. Fold changes were calculated using the 2‐ΔΔCt method.^37^

### RNA‐Seq and pathway analysis

Three biological replicates of DDCs (transduced with LV‐NS or LV‐138) were induced in osteogenic media, and RNA was extracted from day 2 and day 7 cultures. RNA samples were prepared for sequencing with the Illumina oligo‐dT priming system (Illumina, San Diego, CA, USA) on an Illumina HiSeq 3000. RNA‐Seq reads were demultiplexed with Illumina's bcl2fastq2 and then aligned to the Ensembl release 76 top‐level assembly with STAR version 2.0.4b.^38^ Gene counts were imported into the R/bioconductor package EdgeR^39^, ^40^; the trimmed mean of *M*‐values normalization (TMM) method was used to adjust for differences in effective library size across all samples. Results were filtered for genes with Benjamini‐Hochberg FDR adjusted *p*‐values ≤0.05. The R/bioconductor package heat map3^41^ was used to display heat maps of all significant genes. Global perturbations in known KEGG pathways were detected using the R/bioconductor package GAGE.^42^ The R/bioconductor package Pathview^43^ was used to generate annotated pathway maps on perturbed KEGG signaling and metabolism pathways. Raw and processed data from this study have been submitted to the Gene Expression Omnibus (GEO; National Center for Biotechnology Information, U.S. National Library of Medicine, Bethesda, MD, USA) repository and assigned the accession code, GSE109108.

### Western blot

Cell lysates were prepared with RIPA buffer (50mM Tris‐HCl, pH 8.0, 150mM NaCl, 1% Triton‐X‐100, 0.1% sodium dodecyl sulfate, and 0.5% sodium deoxycholate) supplemented with 1× cOmplete protease inhibitor cocktail (Roche Diagnostics, Mannheim, Germany) and phosphatase inhibitor cocktail (Pierce, Rockford, IL, USA). Total protein concentration was measured using the Bio‐Rad protein assay kit (Bio‐Rad Laboratories, Hercules, CA, USA). Proteins were resolved by SDS‐PAGE and transferred onto a polyvinylidene fluoride membrane. Protein‐containing membranes were blocked with Odyssey Blocking Buffer (LI‐COR Biosciences, Lincoln, NE, USA) containing 0.1% Tween 20. Western blots were performed with mouse anti‐β‐actin antibodies (Abcam ab6267; Abcam, Cambridge, UK) and either rabbit anti‐RUNX2 (Cell Signaling Technology #8486; Cell Signaling Technology, Inc, Danvers, MA, USA), rabbit anti‐osteocalcin (Abcam ab93876), or rabbit anti‐RhoC (Cell Signaling Technology #3430) primary antibodies. Primary antibodies were used at a 1:1000 dilution except for anti‐osteocalcin (1:400 dilution). Data were normalized to β‐actin, a method used in other studies to quantify differences in levels of RhoC.^33^, ^34^ Secondary antibodies (IRDye 800CW‐labeled anti‐rabbit; IRDye 680RD‐labeled anti‐mouse; LI‐COR Biosciences) were used at a 1:10,000 dilution following the manufacturer's recommendation. Resulting band intensity was calculated using the LI‐COR Odyssey software (LI‐COR Biosciences).

### Cell proliferation assay

Lentiviral transduced DDCs were seeded in 96‐well plates at a density of 1 × 10^4^ cells/well. Cell proliferation was measured 24 hours later by using the BrdU Cell Proliferation ELISA Kit following the manufacturer's protocol (Abcam ab126556). Data were quantified using a spectrophotometer set at a dual wavelength of 450 nm/550 nm.

### In vitro scratch assay

Nontransduced, LV‐NS, or LV‐138‐transduced DDCs were seeded onto 12‐well plates at a density of 2 × 10^5^ cells/well. After 48 hours (approximately 80% confluency), a scratch (wound) was created across the center of each well using a sterile 1‐mL pipette tip and immediately photographed. This was designated time zero. Cells were cultured in growth media; additional images were taken 24 hours and 48 hours later. Cells within the scratched area were monitored at each time point and analyzed with Image J software (Version 1.5.1, https://imagej.nih.gov/ij/download.html) using the “Analyze Particles” command setting. The relative coverage (ie, the area covered by cells divided by the total scratch wound area) was calculated at 48 hours.

### Cytoskeletal F‐actin staining

Nontransduced, LV‐NS, or LV‐138 DDCs were seeded in 8‐well chamber slides at a density of 1 × 10^4^ cells/well and cultured in osteogenic medium for 48 hours. Cells were fixed with 4% paraformaldehyde for 20 min and incubated with a Phalloidin‐iFluor 488 reagent (Cytopainter; Abcam ab176753) for 1 hour that selectively binds to intracellular F‐actin filaments. Cells were washed 3 times in 1× PBS followed by the addition of a fluoro‐gel mounting medium containing DAPI (Electron Microscopy Sciences, Hatfield, PA, USA). Cells were imaged with a fluorescent microscope at excitation/emission = 493 nm/517 nm.

### miRNA target prediction

A search for predicted miR‐138 target mRNAs was performed using the databases TargetScan (http://www.targetscan.org/),^44^ miRTarBase (http://mirtarbase.mbc.nctu.edu.tw/),^45^ and miRDB (http://www.mirdb.org/).^46^

### Overexpression and knockdown of RhoC

Lentiviral vector pReceiver‐Lv105 containing human RhoC mRNA (NM_001042679.1) (LV‐RhoC) was obtained from GeneCopoeia (EX‐I0317‐Lv105; GeneCopoeia, Inc, Rockville, MD, USA). An empty control vector for pReceiver‐Lv105 (LV‐CTL) was purchased from the same company (EX‐NEG‐Lv105). Lentiviral vectors carrying RhoC ShRNA (TRCN0000307711) (LV‐ShRhoC), or nontarget ShRNA Control (SHC016‐1EA) (LV‐ShCTL) were purchased from Sigma‐Aldrich (St Louis, MO, USA). Lentiviral stocks were prepared as previously described^36^ and were titered using the Lenti‐X p24 rapid titer ELISA (Clontech Laboratories). RhoC overexpression or knockdown in DDCs was confirmed by qPCR and Western blot. For the rescue experiments, DDCs were first transduced with LV‐NS or LV‐138 at an MOI of 20 in growth medium containing 100 μg/mL protamine sulfate for 24 hours. Three days after transduction, DDCs were reseeded and transduced again with LV‐RhoC or LV‐CTL at an MOI of 20 in growth medium containing 100 μg/mL protamine sulfate for 24 hours. Three days after this second transduction, DDCs were reseeded to carry out proliferation assays, F‐actin staining, cell‐migration assays, or osteogenesis assays as already described. For RhoC knockdown experiments, DDCs were transduced with LV‐ShRhoC; 3 days later, cells were reseeded for assays to examine proliferation, F‐actin staining, and osteogenesis.

### Osteogenesis in 3D bone scaffolds

Human cancellous bone was harvested from OA knee joints following total knee replacement surgery, washed thoroughly with Dulbecco's PBS (DPBS), and decalcified by using a mild immunocal formic acid decalcifier solution (1414‐X StatLab, McKinney, TX, USA) for 3 days. Partially decalcified cancellous bone was cut into 5 mm^3^ pieces and further decalcified for 3 days. Resulting decalcified bone “scaffolds” were washed thoroughly in DPBS (3‐ × 6‐hour washes), immersed in an antibiotic solution (10,000 unit/mL penicillin, 10,000 μg/mL streptomycin) for 6 hours followed by additional washes in DPBS (3 × 30 min). Bone scaffolds were air‐dried and stored at room temperature before use. Scaffolds were imaged by micro‐CT using a Scanco μCT40 scanner (Scanco Medical AG, Brüttisellen, Switzerland) to establish a baseline density prior to osteogenesis. Scans were completed at 45 kVp and 177 μA with 300‐ms integration time at an effective voxel size of 6 μm. DDCs (5 × 10^5^) transduced with LV‐NS, LV‐138 + LV‐RhoC, or LV‐138 + LV‐CTL were seeded onto each scaffold and cultured in growth medium for 48 hours prior to addition of osteogenic culture medium. Micro‐CT scans of day 28 scaffolds were collected, and using Scanco (V6.5) software, the morph tool was used to define a region of interest and contours were drawn every 20 slices. A threshold value of 180 was set for the evaluation of all scanned images. VOX‐BV (voxel of bone volume) and VOX‐TV (voxel of total volume) values were used to calculate bone volume relative to total volume (BV/TV). BMDs within the scaffolds were also calculated. 3D images and movies were created using DRAGONFLY software (Object Research Systems, Inc, Montreal, Canada). An additional histological analysis of scaffolds was carried out following Alizarin Red staining of scaffold paraffin sections.

### Statistics

All experiments were carried out in triplicate with DDCs derived from at least three independent human OA articular cartilage specimens. Data are presented as means ± SD; statistical comparisons were made using an unpaired Student's *t* test. In the case of cell proliferation, in vitro scratch assays and 3D osteogenesis assays, multiple comparisons were made using one‐way (ANOVA. Probability values were considered statistically significant at *p* ≤ 0.05.

## Results

### Osteogenic differentiation of DDCs

Dedifferentiation of primary chondrocytes was confirmed at P4 by detection of a fibroblastic phenotype (results not shown) and increased expression levels of *COL1A1* relative to *COL2A1* (Supplemental Fig. 1). Osteogenic differentiation of nontransduced DDCs was achieved as indicated by increased expression in *RUNX2*, osterix (OSX), osteocalcin (OCN), bone sialoprotein (BSP), osteopontin (OPN), alkaline phosphatase (ALP), and matrix metalloproteinase‐13 (MMP13) (Supplemental Fig. 2*A–C*). Enhanced ALP activity, matrix calcification, and hydroxyapatite formation were also shown (Supplemental Fig. 2*D–F*). Successful osteogenesis was also confirmed in DDC cultures transduced with lentivirus overexpressing a control NS RNA (Supplemental Fig. 3*A–F*). This shows that lentiviral transduction does not negatively affect the ability of DDCs to differentiate. Effects of miR‐138 overexpression in DDCs were therefore always compared with DDCs transduced with LV‐NS.

### miR‐138 inhibits osteogenic differentiation of DDCs

**Figure 1:**
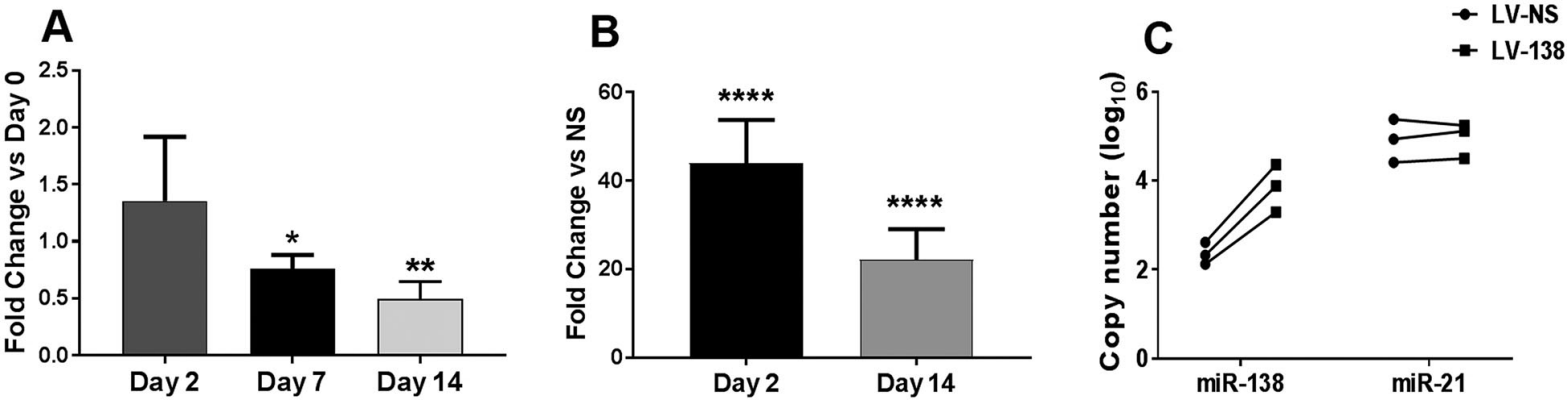
Expression of miR‐138 in dedifferentiated chondrocytes (DDCs). Endogenous expression of miR‐138‐5p in DDCs at day 2, 7, or 14 of osteogenic induction (*A*). Overexpression of miR‐138‐5p in DDCs transduced with LV‐138 at either day 2 or day 14 of osteogenic differentiation (*B*). Following normalization to RNU44, fold change expression of miR‐138‐5p was calculated relative to day 0 (*A*) or LV‐NS at each time point (*B*). Data in (*A*) and (*B*) are expressed ± SD; *n* = 3. *p < 0.05; **p < 0.01; ****p < 0.0001. Quantified copy numbers of miR‐138‐5p or miR‐21‐5p in DDCs transduced with either LV‐NS or LV‐138 (*n* = 3) (*C*).

**Figure 2:**
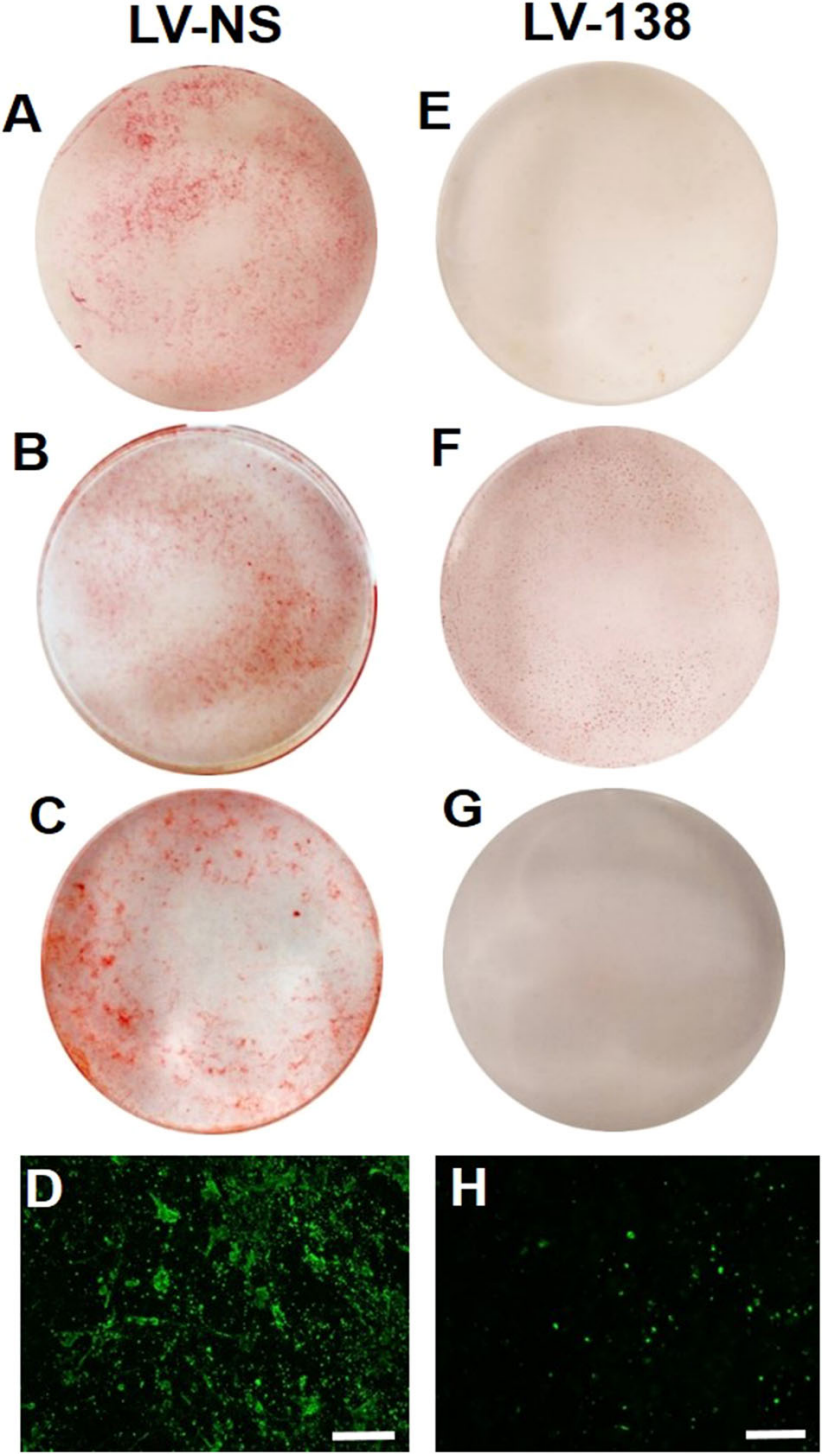
Inhibition of matrix mineralization by miR‐138. Alizarin Red stained cultures of osteogenic‐induced dedifferentiated chondrocytes (DDCs) transduced with either LV‐NS (*A–C*) or LV‐138 (*E–G*) at day 14. Images are from three independent DDC cultures derived from articular cartilage of different patients with osteoarthritis: (*A*) and (*E*): patient 1; (*B*) and (*F*): patient 2; (*C*) and (*G*): patient 3. Hydroxyapatite staining in DDC cultures transduced with either LV‐NS (*D*) or LV‐138 (*H*). (*D*) and (*H*) are representative images of three independent experiments. Scale bars = 200 μm.

**Figure 3:**
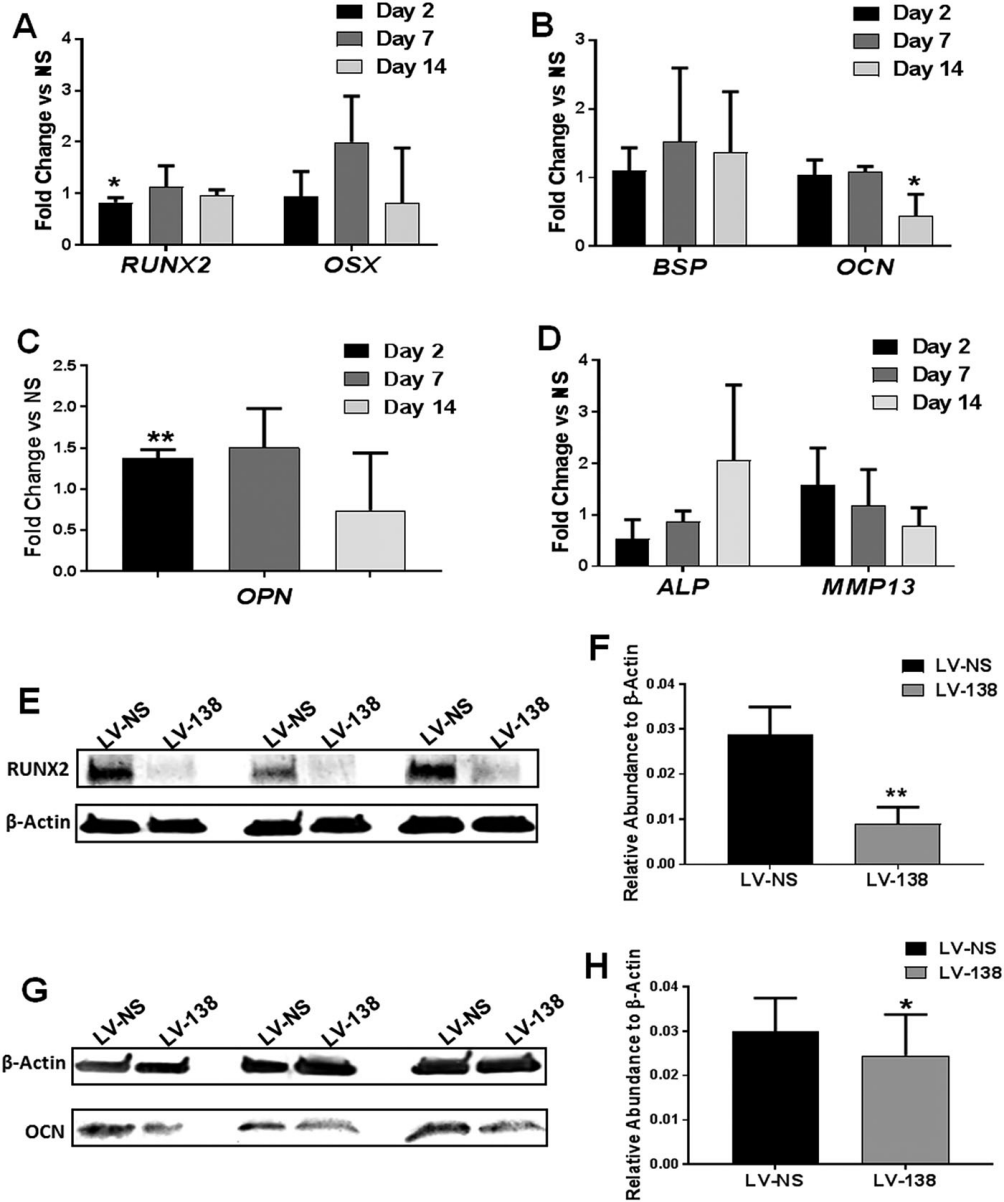
Effects of miR‐138 on osteogenic gene and protein expression. Fold change differences in gene expression in LV‐138‐tranduced cultures compared with LV‐NS control cultures at day 2, 7, or 14 following osteogenic differentiation (*A–D*). Note that the unpaired *t*‐test was used to compare gene expression in miR‐138‐transduced cells compared with nonsilencing control cells at each individual time point. Western blots show decreased expression of RUNX2 (*E, F*) or osteocalcin (OCN) (*G, H*) protein following overexpression of LV‐138 after 4 days or 14 days of osteogenic induction, respectively. β‐actin = 42 kDa; RUNX2 = 57 kDa; OCN = 11 kDa. RUNX2 and OCN Western blots are quantified in (*F*) and (*H*), respectively. Data in (*F*) and (*H*) are expressed ± SD; *n* = 3. *p < 0.05; **p < 0.01.

Endogenous expression of miR‐138 was found to decrease over time during DDC osteogenic differentiation (Fig. 1 *A*). This finding provided the rationale to overexpress miR‐138 to determine its effects on regulating osteogenesis of DDCs. We first confirmed overexpression of miR‐138 in primary DDCs over time in culture. Figure 1 *B* shows increased miR‐138 levels in DDCs at day 2 and day 14 of differentiation when compared with LV‐NS‐transduced cells at the same time point, albeit overexpression decreased over time in culture. Figure 1 *C* shows that levels of overexpression were within physiological range (ie, lower than endogenous levels of miR‐21, a known highly expressed miRNA). Overexpression of miR‐138 inhibited matrix mineralization as shown by a substantial decrease in both Alizarin Red and hydroxyapatite staining compared with LV‐NS transduced cultures at day 14 (compare Figs. 2 *A*–*D* with Figs. 2 *E*–*H*). Interestingly, low to negligible changes in osteogenic gene expression were found in cultures transduced with LV‐138 compared with LV‐NS. Among these changes, only *RUNX2* and *OCN* gene expression showed a significant decrease in fold change expression at day 2 or day 14, respectively (Fig. 3 *A*,*B*). A modest increase in osteopontin gene expression was found at day 2 (Fig. 3 *C*) while no significant changes in alkaline phosphatase or MMP‐13 gene expression was found at any time point (Fig. 3 *D*). However, a more apparent and significant decrease in RUNX2 (Fig. 3 *E*,*F*) and OCN (Fig. 3 *G*,*H*) protein levels were found following miR‐138 overexpression at day 4 or day 14 of osteogenic differentiation, respectively.

### miR‐138 modulates cell proliferation, cell morphology, and F‐actin formation

**Figure 4:**
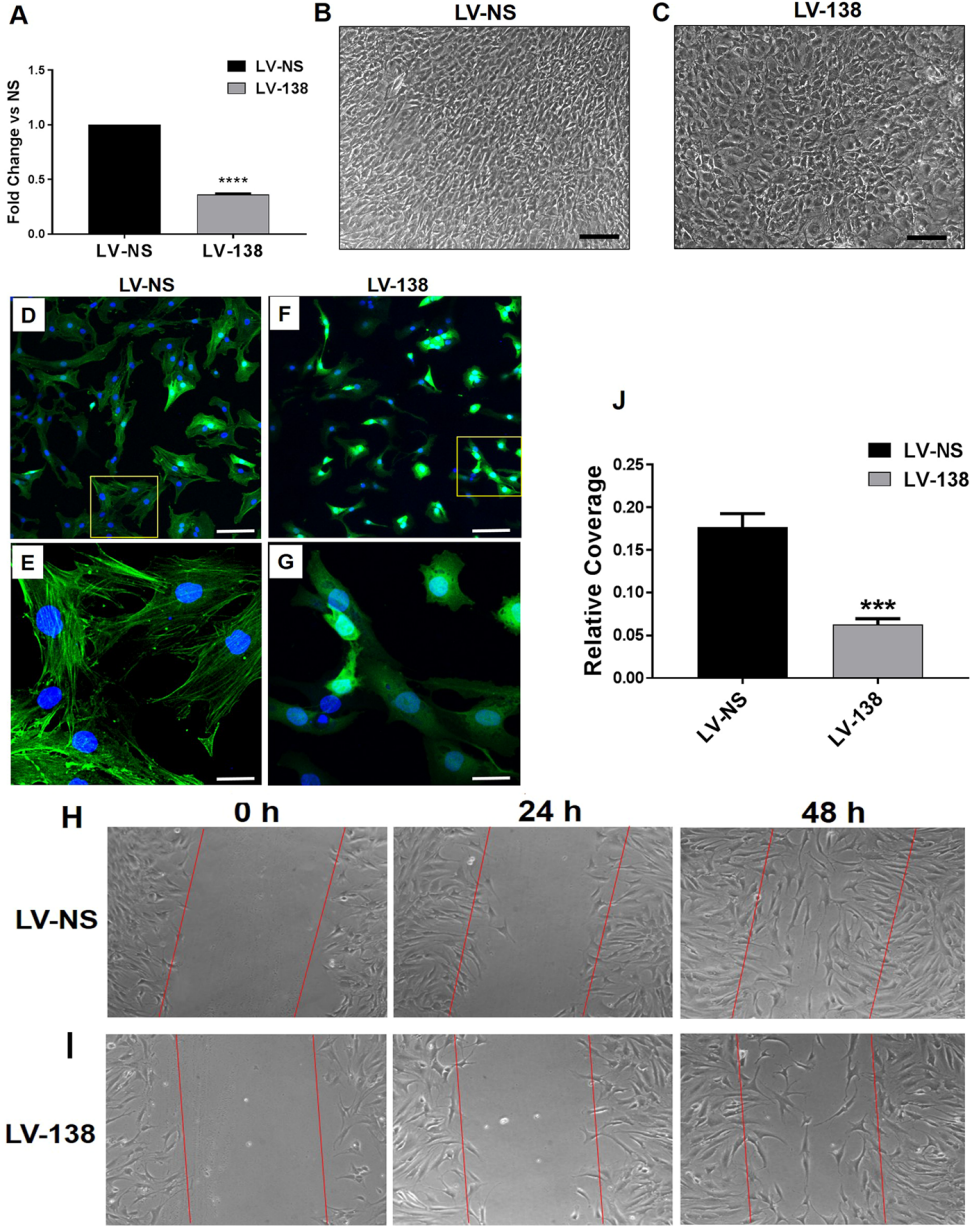
Effects of miR‐138 on cell proliferation, morphology, migration, and the actin cytoskeleton. Fold change difference in cell proliferation of LV‐138‐transduced DDCs compared with cells transduced with LV‐NS (*A*). Light microscopic images of LV‐NS (*B*) or LV‐138‐transduced dedifferentiated chondrocytes (DDCs) (*C*) following 7 days in osteogenic medium. Representative confocal microscopy images shows localization of F‐actin in DDCs transduced with either LV‐NS (*D,E*) or LV‐138 (*F,G*) after 48 hours in osteogenic medium. Yellow‐boxed areas in (*D*) and (*F*) are shown at higher magnification in (*E*) and (*G*), respectively. Light microscopy images show migration of DDCs transduced with LV‐NS (*H*) or LV‐138 (*I*) into the scratch wound area over a 48‐hour period. Quantification of cells covering the scratch wound area at 48 hours is shown in (*J*). Scale bars in (B,C) = 100 μm; scale bars in (*D,F*) = 200 μm; scale bars in (*E,G*) = 50 μm. Data are expressed ± SD; *n* = 3. ***p < 0.001; ****p < 0.0001.

We consistently found lower levels of RNA extracted from LV‐138‐transduced cultures compared with control cells, suggesting that cell proliferation may be affected. We found that overexpression of miR‐138 significantly inhibited cell proliferation by over 60% after 24 hours in culture when compared with LV‐NS transduced control cells (Fig. 4 *A*). Following 7 days in osteogenic medium, differences in cell shape were observed in LV‐138 cultures when compared with LV‐NS‐transduced cells (Fig. 4 *B*,*C*). Changes in cell morphology in these high‐density day‐7 osteogenesis cultures suggested that cell polarity may be disrupted, which could be caused by alterations in the cell cytoskeleton as well as by abnormalities in cell movement/migration. Staining of DDCs with a fluorescently labeled phalloidin conjugate showed differences in cell shape at lower magnification due to miR‐138 over‐expression where cells were less spread‐out (compare Fig. 4 *D* and Fig. 4 *F*). At higher magnification, abundant F‐actin filaments were found in cells transduced with LV‐NS (Fig. 4 *E*), but not in DDCs transduced with LV‐138 (Fig. 4 *G*). Figure 4 *H–J* shows that miR‐138 significantly inhibited the number of cells found in the scratch wound site over a 48‐hour period when compared with DDCs transduced with LV‐NS. Though this suggests that cell migration/movement has been inhibited by miR‐138 overexpression, it should be noted that these findings could be caused, in part, by the inhibitory effect of miR‐138 on cell proliferation.

### Identification of RhoC as a target gene of miR‐138 in DDCs

**Figure 5:**
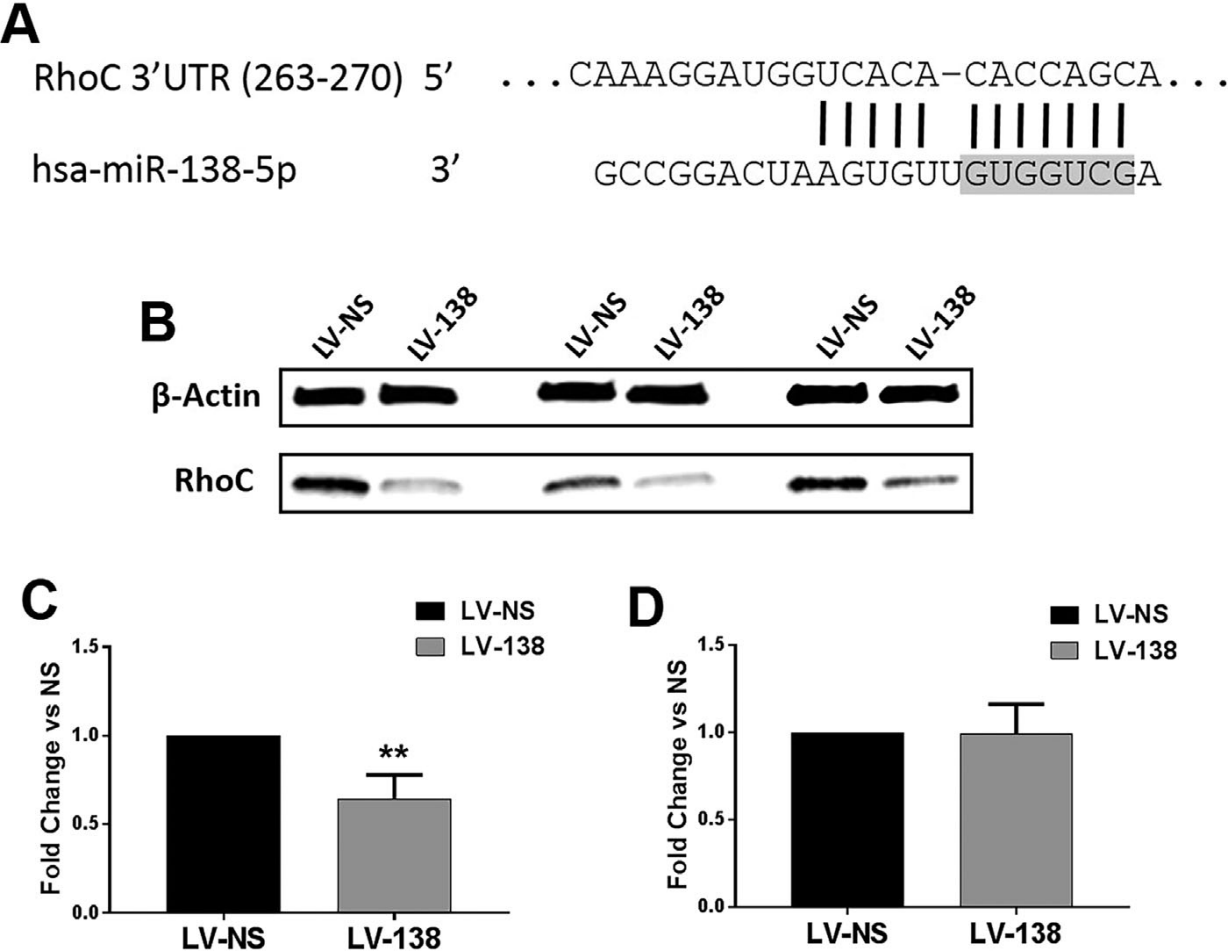
RhoC is a target of miR‐138. The 3'UTR of human RhoC mRNA contains one miR‐138 binding site. The seed sequence of miR‐138 is shaded grey (*A*). Western blot of three biological replicate samples showing reduced RhoC expression in DDCs following transduction with LV‐138 (*B*). β‐actin = 42 kDa; RhoC = 21 kDa. Fold change decrease in RhoC protein expression is quantified in (*C*). RhoC gene expression in LV‐138‐transduced dedifferentiated chondrocytes (DDCs) was compared with expression in DDCs transduced with LV‐NS (*D*). Data are expressed ± SD; *n* = 3. **p < 0.01.

A search for predicted miR‐138 target genes was performed using three web‐based servers: TargetScan (http://www.targetscan.org/),^44^ miRTarBase (http://mirtarbase.mbc.nctu.edu.tw/),^45^ and miRDB (http://www.mirdb.org/).^46^ One highly scored target gene identified by all three programs was the small GTPase, RhoC (Fig. 5 *A*). We pursued this target gene further, given that RhoC has been shown to regulate cell proliferation, migration, and morphology.^32^, ^34^, ^47^ Indeed, we found decreased levels of RhoC in DDCs transduced with miR‐138 when compared with LV‐NS‐transduced cells (Fig. 5 *B*,*C*). No difference in RhoC gene expression was found (Fig. 5 *D*), indicating that miR‐138 inhibits RhoC at the level of translation. Of note, we did not perform luciferase reporter assays (ie, using constructs containing the 3'UTR sequence of RhoC or a mutant version thereof) as an additional approach to confirm that RhoC is a target of miR‐138 because this has already been shown in other studies using cancer cells.^48^, ^49^

### RNA‐Seq and pathway analysis

**Figure 6:**
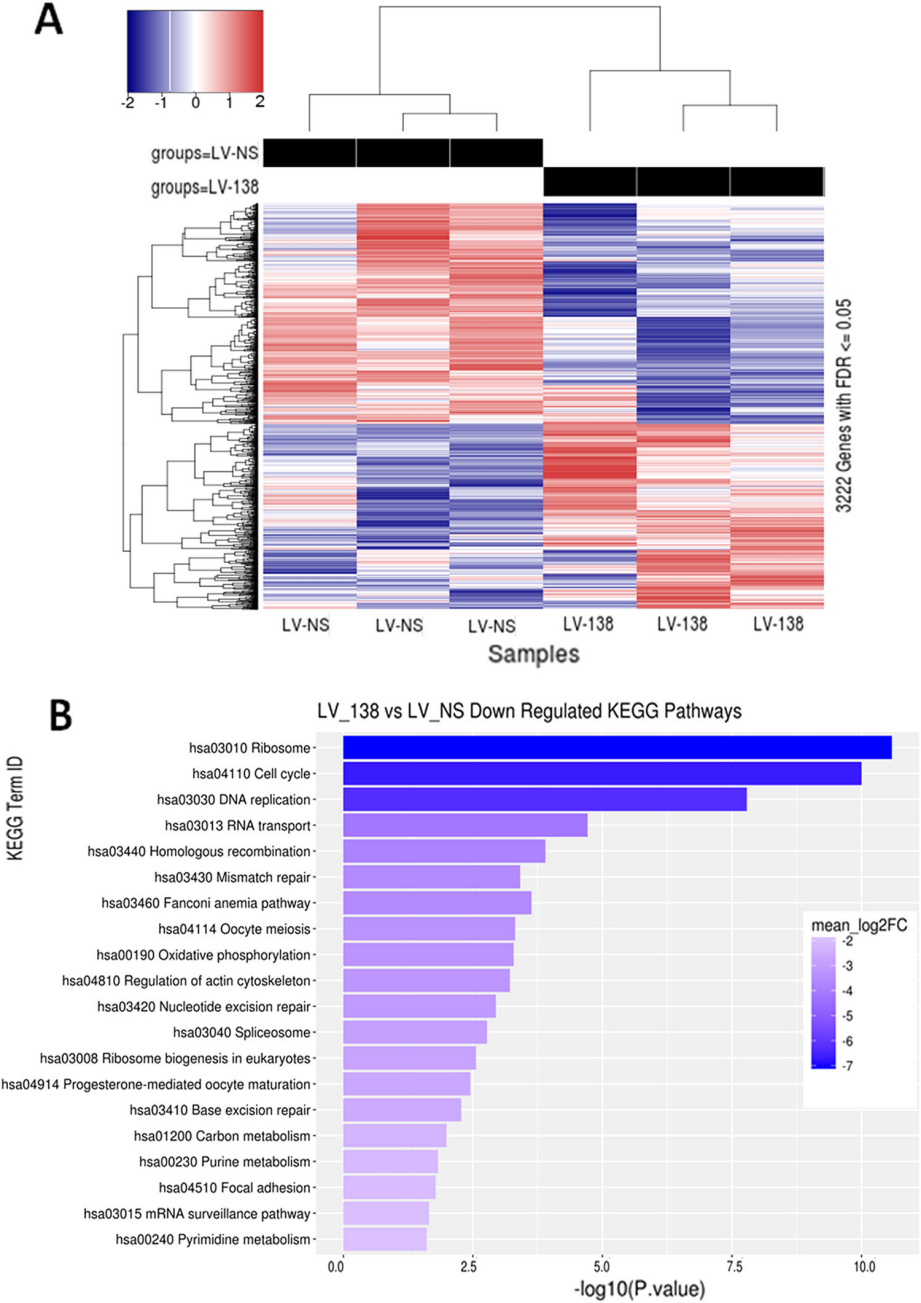
Statistically significant gene‐ and system‐level effects of miR‐138 overexpression. Heat map of 3222 genes, organized by hierarchical clustering, with Benjamini‐Hochberg FDR adjusted *p*‐values <0.05 (*A*). Bar plot of the top 20 significantly downregulated KEGG signaling and metabolism pathways affected by miR‐138 overexpression. Significance has been −log10 scaled for readability and each term has been color‐coded to show the extent of mean log 2 fold‐change downregulation (*B*).

RNA‐Seq and KEGG pathway analysis confirmed some of the changes we observed following miR‐138 overexpression in DDCs. The heat map in Fig. 6 *A* shows differences in levels of over 3000 genes and, importantly, significant differences were found based on miR‐138 overexpression at day 7 of osteogenesis. Most genes that showed statistically significant expression changes with FDR adjusted *p*‐values ≤0.05 had moderate‐to‐low log 2 fold change differentials. This suggested that overall differences in observed phenotypes were likely the combination of small, additive effects of biological pathways. Gene‐expression data were further interrogated for global level changes in known KEGG signaling and metabolism pathways; Fig. 6 *B* shows the top 20 most significantly downregulated pathways. Of note are the significant downregulation of pathways related to cell cycle (Supplemental Fig. 4*A*) and regulation of the actin cytoskeleton (Supplemental Fig. 4*B*), which support our findings showing that miR‐138 inhibits cell proliferation and F‐actin polymerization in DDCs.

### Effects of RhoC overexpression in miR‐138 transduced DDCs

**Figure 7:**
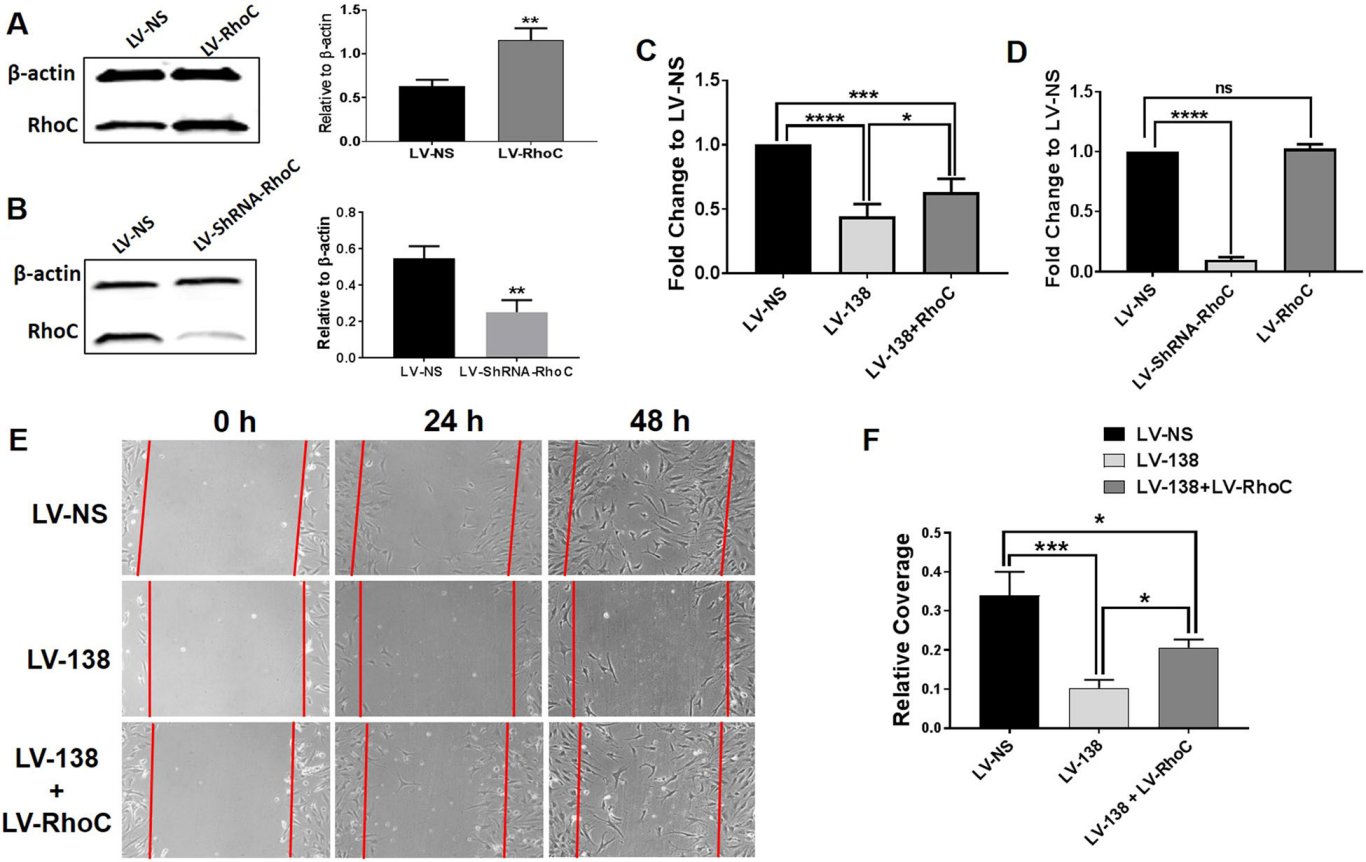
Modulation of dedifferentiated chondrocyte (DDC) proliferation and migration by altering RhoC expression. Western blots and corresponding graphs quantifying band intensities shows overexpression of RhoC (*A*) or knockdown of RhoC (*B*) in DDCs following 4 days of transduction with RhoC mRNA or shRNA RhoC‐expressing lentivirus. DDC proliferation was induced by RhoC overexpression in LV‐138 transduced cells at 24 hours compared with LV‐138 transduced DDCs (*C*). Knockdown of RhoC alone showed a significant decrease in proliferation compared with control cells, but no differences were found when RhoC alone was overexpressed (*D*). Enhanced DDC migration into scratch wound area after 48 hours of transduction with LV‐138 + LV‐RhoC when compared with LV‐138 transduced DDCs (*E*). Quantification of cells covering the scratch wound area at 48 hours (*F*). Scale bars in (*D*) = 10 μm. Data are expressed ± SD; *n* = 3. *p < 0.05; ***p < 0.001; ****p < 0.0001.

To attempt to link the cellular effects of miR‐138 to RhoC inhibition, a series of rescue experiments were designed to overexpress RhoC in LV‐138‐transduced cells. Specifically, the effects of RhoC overexpression on potentially attenuating the inhibitory actions of miR‐138 on cell proliferation, F‐actin polymerization, cell migration, and osteogenesis were investigated. We first confirmed that the overexpression of RhoC protein could be induced in DDCs following transduction with LV‐RhoC (Fig. 7 *A*). Figure 7 *C* shows a modest yet significant increase in DDC proliferation in miR‐138‐transduced cultures overexpressing RhoC. As an additional control, we also knocked down RhoC alone (Fig. 7 *B*), and found substantial inhibition of DDC proliferation when compared with control cultures (Fig. 7 *D*). No differences in cell proliferation were found when RhoC alone was overexpressed (Fig. 7 *D*). RhoC overexpression in LV‐138 transduced cells was found to increase the number of cells within the scratch wound area (Fig. 7 *E*,*F*).

**Figure 8:**
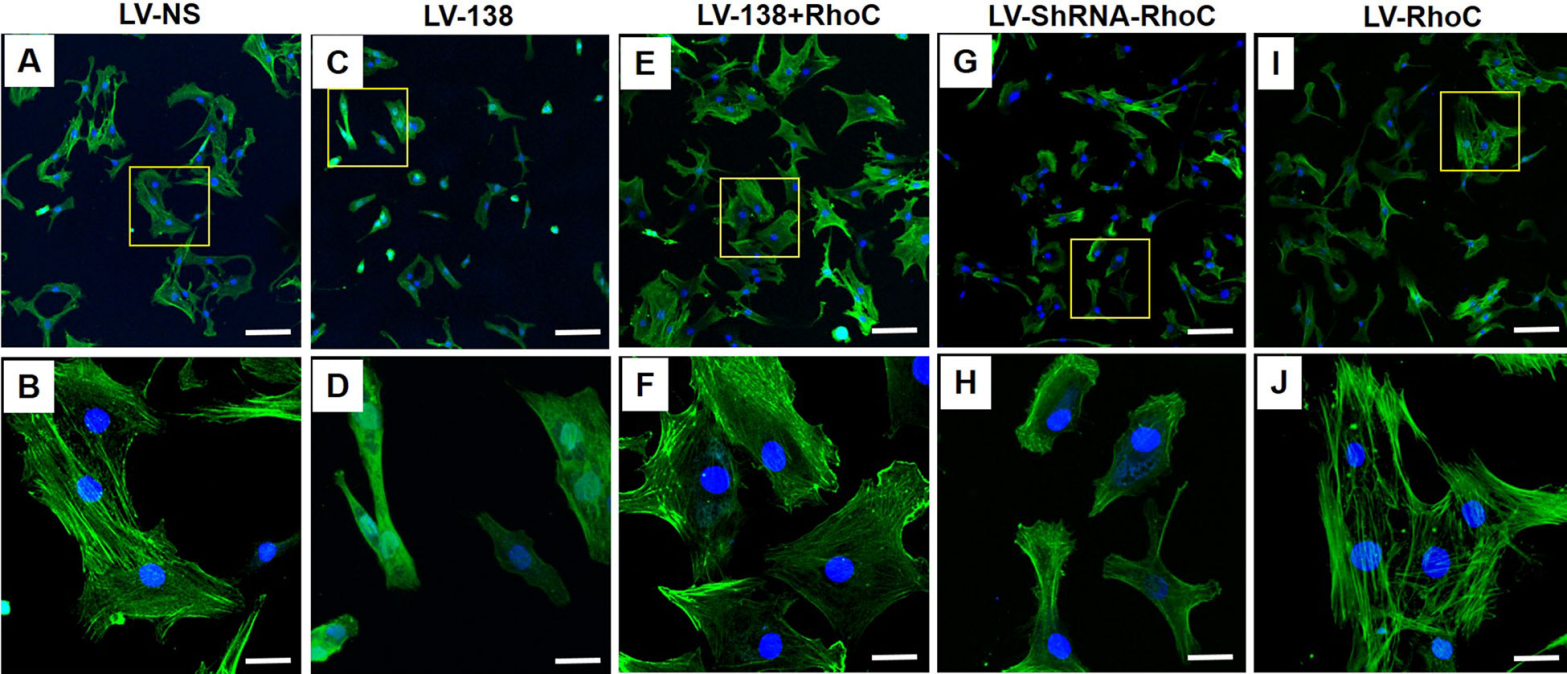
Effects of modulating RhoC expression on the formation of F‐actin in DDCs. Representative confocal microscopy images shows localization of F‐actin in DDCs transduced with LV‐NS (*A,B*), LV‐138 (*C,D*), LV‐138 + RhoC (*E,F*), LV‐ShRNA‐RhoC (*G,H*), or LV‐RhoC (*I,J*) after 48 hours in osteogenic medium. Yellow‐boxed areas in (A), (C), (E), (G), and (I) are shown at higher magnification in (B), (D), (F), (H), and (J), respectively. Scale bars in (*A*), (*C*), (*E*), (*G*), and (*I*) = 200 μm; scale bars in (*B*), (*D*), (*F*), (*H*), and (*J*) = 50 μm.

With respect to F‐actin formation, a partial rescue in cell shape and actin formation was observed in DDCs cotransduced with LV‐138 and LV‐RhoC (Fig. 8 *E*,*F*) when compared with DDCs transduced with LV‐138 alone (Fig. 8 *C*,*D*). Specifically, the overall cell shape based on RhoC overexpression (Fig. 8 *E*) resembled that of the LV‐NS control cells (Fig. 8 *A*), whereby cells were more spread‐out when compared with LV‐138‐transduced cells (Fig. 8 *C*). Importantly, higher magnification images showed the formation of some F‐actin polymers based on RhoC overexpression (Fig. 8 *F*) when compared with LV‐138 transduced cells, where none was found (Fig. 8 *D*). Interestingly, when RhoC expression alone was knocked down, clear differences were noted in cell shape (Fig. 8 *G*) when compared with LV‐NS cells (Fig. 8 *A*), whereby cells were smaller and less spread‐out. Alterations in formation of the actin cytoskeleton were also noted, whereby less‐pronounced F‐actin filaments were found, with what appears to be some fragmented F‐actin present around the periphery of the cells (compare Fig. 8 *B* and 8 *F*). No obvious differences in cell shape or F‐actin formation were noted when RhoC alone was overexpressed in DDCs (compare Fig. 8 *A*,*I*, and Fig. 8 *B*,*J*).

**Figure 9:**
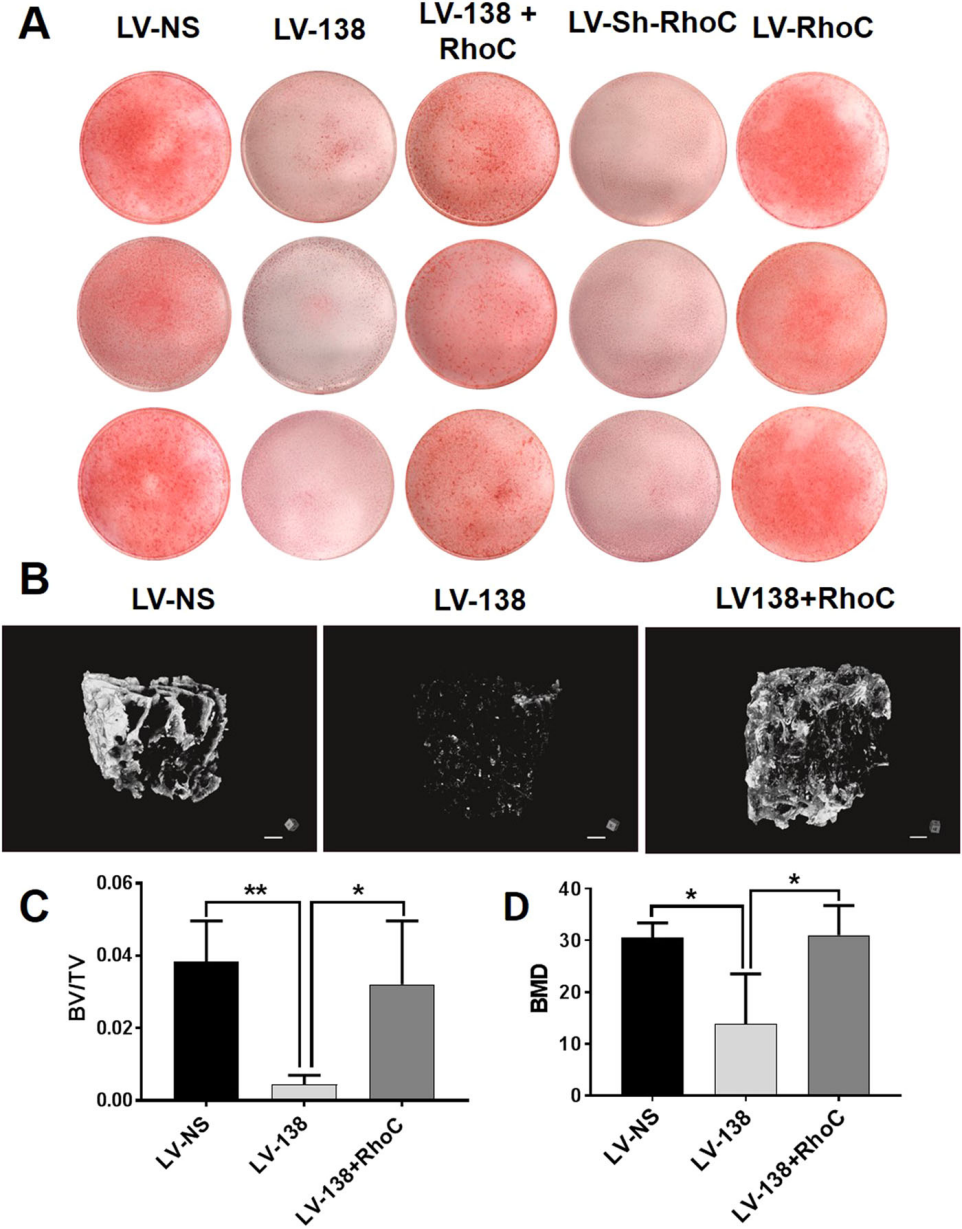
RhoC overexpression rescues miR‐138‐induced inhibition of osteogenesis. Alizarin Red stained cultures at day 14 of dedifferentiated chondrocyte (DDC) osteogenic induction shows enhanced mineralization induced by cotransduction of cells with LV‐138 and LV‐RhoC (*A*). Additional controls (RhoC knockdown alone; LV‐Sh‐RhoC and RhoC overexpression alone; LV‐RhoC) were also included (*A*). Representative micro‐CT images of human decalcified, decellularized bone scaffolds seeded with DDCs transduced with either LV‐NS, LV‐138 + LV‐CTL (empty virus to control for LV‐RhoC overexpression) or LV‐138 + LV‐RhoC (*B*). Quantification of bone volume relative to total volume and BMD is shown in panels (*C*) and (*D*), respectively. BV/TV and BMD data are expressed ± SD; *n* = 4. *p < 0.05; **p < 0.01. Scale bars in (*B*) = 1 mm.

Importantly, the suppressive effects of miR‐138 on osteogenic differentiation of DDCs were also rescued by RhoC as shown by increased Alizarin Red staining at day 14 (Fig. 9 *A*). In addition, knockdown of RhoC alone also caused substantial inhibition of osteogenic differentiation, further confirming the importance of this small GTPase in regulating osteogenesis in vitro (Fig. 8 *A*). No apparent changes in differentiation were noted when RhoC alone was overexpressed (Fig. 8 *A*). We also established a more physiologically relevant 3D system to study bone formation by utilizing human decalcified, decellularized human bone scaffolds seeded with DDCs. Representative micro‐CT images in Fig. 9 *B* show an expected decrease in mineralized bone formation by LV‐138‐transduced DDCs compared with LV‐NS‐transduced DDCs at day 28 of osteogenic induction. Importantly, overexpression of RhoC significantly rescued the inhibitory action of miR‐138 on bone formation (Fig. 9 *B* and corresponding video files). These data correlate with the differences observed in levels of calcification within the scaffolds as shown by Alizarin Red staining of scaffold tissue sections (Supplemental Fig. 5). Quantification of the scanned micro‐CT images showed an expected decrease in bone BV/TV, as well as total BMD in scaffolds seeded with LV‐138‐transduced DDCs (Fig. 9 *C*,*D*). RhoC overexpression resulted in a significant increase in BV/TV and BMD (Fig. 9 *C*,*D*). Altogether, these data strongly suggest that suppression of RhoC is a major mechanism by which miR‐138 inhibits bone formation in DDCs.

## Discussion

Studies have shown how dedifferentiated human chondrocytes (DDCs) can be redifferentiated back toward the chondrocyte lineage,^22^, ^23^, ^24^, ^25^, ^28^ as well as the osteogenic and adipogenic lineages.^29^ To our knowledge, this study is the first to report that DDCs from human osteoarthritic cartilage can be induced toward osteoblast‐like cells that generate a mineralized extracellular matrix. This finding has interesting implications for the use of these cells for the treatment of bone‐related conditions such as fractures. Studies by Barbero and colleagues^29^ also generated clonal DDC cell lines and found that they had chondrogenic potential, but very limited osteogenic potential. This has also been demonstrated in clonally derived cartilage progenitor cells generated by fibronectin selection.^50^ Given these findings, we utilized DDCs without clonal selection to study osteogenesis in vitro, but do acknowledge that we are dealing with a heterogeneous population of cells. For the purpose of these studies, DDCs are being used as an alternative cell source to MSCs to study osteogenic differentiation and determine new miRNA‐mediated mechanisms regulating this process.

We previously reported on differential expression patterns of miRNAs during human embryonic growth‐plate development.^20^ Among those miRNAs that were found to be significantly differentially expressed was miR‐138, which was more highly expressed in differentiated or hypertrophic chondrocytes when compared with progenitor chondrocytes. These expression data do not correlate with that shown by Seidl and colleagues, where expression levels of miR‐138 in differentiated articular chondrocytes were found to be lower than in DDCs.^51^ However, it may be that expression patterns and function of miR‐138 are different in articular versus growth‐plate chondrocytes. Given the fact that growth‐plate development is required for endochondral bone formation, we explored the potential function of miR‐138 in regulating osteogenesis and the mechanisms involved. We found that endogenous expression of miR‐138 during osteogenesis of DDCs decreased over time in culture, and that miR‐138 overexpression caused substantial inhibition of DDC osteogenic differentiation and matrix mineralization, both in monolayer in vitro cultures as well as within human trabecular bone scaffolds ex vivo. We did not attempt to enhance bone formation by transducing DDCs with anti‐miR‐138 because of low endogenous levels of miR‐138 in these cells. However, anti‐miR‐138 has been shown to enhance bone formation in vitro or ex vivo in other studies.^16^, ^17^ Of the osteogenic genes analyzed in our study, a significant decrease in *RUNX2* and *OCN* was observed in response to miR‐138 overexpression, which was confirmed at the protein level. The reason why other osteogenic genes were not found to be significantly suppressed may be because of the heterogeneity in endogenous gene and protein expression that exists in cells extracted from different human osteoarthritic cartilage specimens. Similar to our findings, Eskildsen and colleagues^16^ reported inhibitory effects of miR‐138 on the osteogenesis of human MSCs, but as will be discussed, a different miR‐138‐driven mechanism in DDCs has been discovered in the present study.

We first examined the effects of miR‐138 on cell proliferation because we consistently detected decreased levels of RNA extracted from cultures overexpressing this miRNA. We found proliferation was inhibited, which is in agreement with a number of studies in the cancer field, where miR‐138 has been shown to decrease the proliferation of various tumor cells.^18^ In published studies reviewed by Sha and colleagues,^18^ it is apparent that miR‐138 can function to suppress a number of different target genes related to proliferation. This is not surprising given the fact that miRNAs can function to suppress many targets at the posttranscriptional level in any given cell type.^52^ KEGG analysis of our RNA‐Seq data (Supplemental Fig. 4*A*) points to the downregulation of a number of genes and pathways associated with cell‐cycle regulation, thereby suggesting that miR‐138 is likely targeting proliferation‐related genes either directly or indirectly in DDCs.

In addition to inhibiting proliferation, an apparent reduction in F‐actin polymerization was found in miR‐138‐transduced DDCs when compared with control cells. In agreement with this finding, KEGG analysis of our RNA‐Seq data showed downregulation of a number of genes and pathways associated with the regulation of the actin cytoskeleton (Supplemental Fig. 4*B*). We then predicted, given alterations in the cellular cytoskeleton, that the ability of the cells to migrate may be inhibited. Indeed, a lower number of LV‐138 transduced cells were found within the scratched area after 48 hours, suggesting defects in cell movement/migration. However, in these in vitro scratch assays, we acknowledge that part of the reason we observed lower cell numbers in the scratched area may also be based on the inhibitory effects on cell proliferation. However, there are a number of studies reporting inhibitory effects of miR‐138 on cell migration and invasion in the cancer field, some of which are referenced here.^18^, ^53^, ^54^, ^55^, ^56^, ^57^ Of these studies, Yu and colleagues recently reported that miR‐138 modulates prostate cancer cell migration and invasion by negatively regulating the Wnt/β‐catenin pathway,^56^ a pathway that is also well‐known to be important for osteoblast differentiation.^58^ Furthermore, cell invasion was found to be inhibited by the ability of miR‐138 to target FAK.^57^ Interestingly, FAK, which is important in regulating cell movement, was also identified to be a miR‐138 target gene by Eskildsen and colleagues, who reported inhibition of MSC osteogenesis by miR‐138.^16^ Taken together, it is likely that in DDCs overexpressing miR‐138, a number of different target genes and pathways known to regulate cell proliferation, migration, and the cytoskeleton are simultaneously being suppressed to some degree, thereby rendering the cells unable to properly differentiate.

To begin to decipher mechanism, computational approaches were taken to identify miRNA target genes that could explain the phenotypic effects induced by miR‐138 overexpression in DDCs. One target gene of interest was the small molecular weight GTPase, RhoC (Ras homolog gene family, member C). The Rho GTPase family comprises 20 members in humans^59^ with reported functions in regulating cell proliferation, movement, differentiation, polarity, and cytoskeletal rearrangement.^60^, ^61^ Most of the functional data has been reported for the family members Rho, Rac, and Cdc42. The Rho subfamily includes the isoforms RhoA, RhoB, and RhoC, which are 84% identical in sequence.^62^ Specifically, there are a number of published reports in the cancer field on the role of RhoC in regulating cell migration, invasion, proliferation, morphology, polarity, or the actin cytoskeleton.^31^, ^32^, ^33^, ^34^, ^48^, ^63^ Therefore, we predicted that the inhibition of DDC proliferation, migration, as well as the altered cell morphology and actin cytoskeleton following miR‐138 overexpression could be based, in part, on decreased expression of RhoC. In cancer cells, RhoC has been identified as a miR‐138 target gene via luciferase reporter assays,^48^, ^49^ and our studies showed that RhoC protein levels were indeed lower in DDCs following miR‐138 overexpression. Specific knockdown of RhoC in cancer cell lines was shown to inhibit cell migration and stress fiber formation.^32^, ^33^, ^34^, ^48^ These findings agree with our data where production of F‐actin was inhibited based on overexpression of miR‐138. We believe that the inability to form F‐actin partly explains the overall difference in morphology in DDCs transduced with LV‐138, where the cells appeared smaller and less able to spread out in culture (Fig. 4 *F* and Fig. 8 *C*) when compared with control DDCs. Also, it has been reported that cell spreading favors osteogenesis.^64^ Therefore, the lack of cell spreading in cells transduced with either LV‐138 or shRNA to knockdown RhoC expression alone can certainly explain why osteogenesis is inhibited in these cultures. Note that the cell morphology changes appear different between LV‐NS and LV‐138 transduced cells following 7 days of osteogenic differentiation (Fig. 4 *B* and *C*) when compared with cells cultured in chamber slides (Fig. 4 *D* and 4*F*). This is because cells are plated at a much higher density for osteogenic differentiation assays in addition to having longer times in culture. The experiments to specifically monitor changes in F‐actin formation were carried out in lower cell densities in chamber slides for shorter periods (24 to 48 hours) to better monitor changes in actin cytoskeleton.

Importantly, we have convincingly shown that overexpression of RhoC in DDCs was able to partially rescue the inhibitory action of miR‐138 on cell proliferation, F‐actin polymerization, and osteogenic differentiation. Although miR‐138 undoubtedly suppresses a number of other target genes at the level of translational inhibition or RNA degradation in DDCs, it is clear that suppression of RhoC is a major mechanism by which miR‐138 can modulate a number of cellular processes leading to inhibition of osteogenic differentiation. It may be that the action of RhoC in regulating cell proliferation and F‐actin polymerization is particularly important in restoring osteogenesis in miR‐138 transduced DDCs. Indeed, our data show that RhoC knockdown alone can significantly inhibit DDC proliferation and alter F‐actin formation (Fig. 7 *D* and Fig. 8 *B*,*H*). Importantly, we also showed that RhoC knockdown alone can significantly inhibit osteogenic differentiation of DDCs (Fig. 9 *A*). A number of studies have reported the importance of inducing actin polymerization to promote progenitor cell differentiation toward the osteoblast lineage, and that RhoA is an important regulator in this process.^64^, ^65^, ^66^, ^67^ Evidence from other reports show that suppression of RhoC in cancer cells either via siRNA or via miR‐138 mimics does not affect expression levels of RhoA.^32^, ^48^ Therefore, it is likely that inhibition of actin polymer formation observed in DDCs in our study is not based on any indirect suppressive effects on RhoA, but rather a direct action of miR‐138 in downregulating RhoC expression.

One bone‐related study reported RhoC as an NFATc1‐responsive gene, yet follow‐up studies showed that *RhoC* null mice did not display a bone phenotype nor did RhoC appear to play a role in osteoclast biology.^68^ The fact that *RhoC* null mice do not display a developmental phenotype suggests some functional redundancy with other Rho family members in vivo.^30^ However, our RhoC knockdown experiments clearly suggest an important role for RhoC in regulating osteoblast differentiation, at least in vitro. Also, when *RhoC* ^‐/‐^ embryonic fibroblasts were cultured in vitro under serum starvation stress, pronounced abnormalities in cytoskeletal structures were found.^30^ Therefore, it is possible that *RhoC* null mice may display a phenotype following an injurious/stress challenge, such as a bone fracture, which requires formation of new tissue via stem/progenitor cell proliferation, migration, and differentiation. Similarly, miR‐138‐1 or miR‐138‐2 null mice do not display a phenotype, which may not be surprising given that in each mouse model, expression of miR‐138 from the other chromosomal location will occur. Whether a miR‐138‐1/2 double knockout model displays a phenotype remains to be examined. However, in many cases, miRNA KO mice do not display any phenotype because of redundancy with other miRNA family members.

Currently, there are no published reports on the role of RhoC in regulating osteoblast differentiation. A study by Montero and colleagues showed that in chick limb development, RhoC had an antichondrogenic effect, whereas impairment of RhoC function resulted in exacerbated ectopic chondrogenesis.^69^ In this study, RhoC was shown to promote the formation of stress fibers and focal adhesion complexes. These findings can explain the chondrogenic inhibitory action of RhoC because it is well‐known that suppression of actin polymerization promotes chondrocyte differentiation by inducing a more‐rounded cell phenotype.^70^, ^71^, ^72^ As already discussed, the opposite is true for osteoblast differentiation, thereby further supporting our proposal that a major effect of RhoC in enhancing osteogenesis, within the context of miR‐138 overexpression, is via induction of F‐actin polymerization, thereby allowing cell spreading. Our data and that of others^16^ show antiosteogenic effects of miR‐138 when overexpressed early in inducible progenitor cells. It will be interesting to determine if miR‐138, when overexpressed at later time points after progenitor cell commitment, can also function to inhibit the osteogenic process. We predict that there may be little to no effect of miR‐138 on bone matrix formation. We are currently designing experimental approaches to address this question that will involve a doxycycline‐inducible system to overexpress miRNAs in vitro at a desired time point of differentiation.^73^

It is becoming apparent in the cancer field that there is a regulatory link between miR‐138 and RhoC. Specifically, the action of miR‐138 in suppressing RhoC has been shown to result in inhibition of cancer cell proliferation, migration, and invasion.^48^, ^49^, ^74^ This makes sense in the context of findings from *RhoC* null mice, whereby RhoC was demonstrated to play a critical role in inducing cancer metastasis.^30^ However, the functional interplay between miR‐138 and RhoC in physiological tissue developmental or repair processes is not yet understood. With respect to downstream mechanism, RhoC has been shown to function via interaction with specific formin proteins. The majority of the formin family of proteins are Rho GTPase effectors that function in regulating actin cytoskeleton structures.^75^ Depending on the cell system, RhoC has been shown to functionally interact with the formins, mDia1, FMNL2, or FMNL3.^32^, ^76^, ^77^, ^78^ It will be interesting in future studies to elucidate which formins play an important role in mediating the actions of RhoC during osteoblast differentiation.

In conclusion, our studies have identified a novel mechanism regulating osteogenesis involving miR‐138‐induced suppression of RhoC. Our data, showing inhibition of osteogenic differentiation by RhoC knockdown alone, also demonstrate an unappreciated functional role for RhoC in regulating osteogenesis. Further studies on how miR‐138/RhoC‐regulated pathways affect osteoblast differentiation may prove to be important for the future development of novel strategies to regulate ectopic bone formation or bone repair processes.

## Supporting information

Supporting SuppVideo S1.

Supporting SuppVideo S2.

Supporting SuppVideo S3.

Supporting Figures S1.
