# Supplementary material for: MicroRNA‐138 Inhibits Osteogenic Differentiation and Mineralization of Human Dedifferentiated Chondrocytes by Regulating RhoC and the Actin Cytoskeleton
Source: JBMR Plus. 2018 Jul 18;3(2):e10071. doi: 10.1002/jbm4.10071 (PMC6383697; doi:10.1002/jbm4.10071)

**Supplemental Figure Legends**

**Supplemental Fig. 1: Expression of *COL1A1* and *COL2A1* in human dedifferentiated chondrocytes.** Primary human chondrocytes from OA articular cartilage were passaged 4-5 times and expression of *COL1A1* and *COL2A1* was assessed. The abundance of each gene (± SD; *n* = 3) was expressed relative to the housekeeping gene, *PPIA* (peptidylprolyl isomerase A).


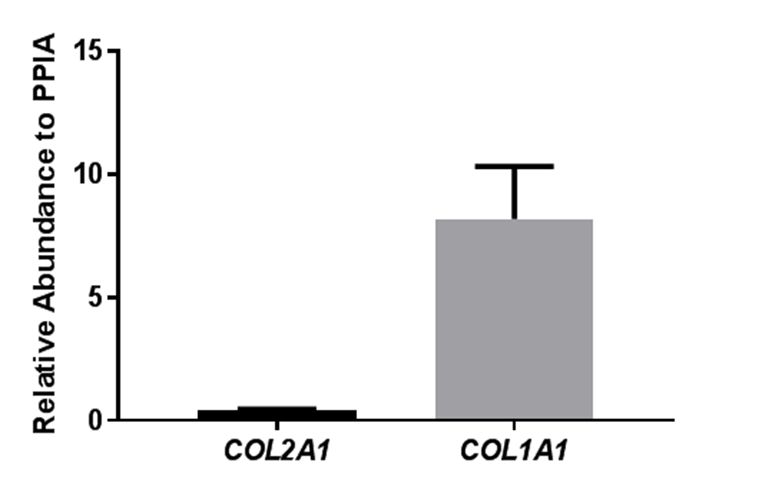


**Supplemental Fig. 2**: **Osteogenic induction of human non-transduced dedifferentiated chondrocytes.** Expression of osteogenic genes (A-C) was assessed at day 2, 7, or 14 after osteogenic induction of DDCs. Each gene was normalized to *PPIA* and then calculated as fold change expression compared to day 0 (± SD; *n* = 3; * *p* < 0.05). Osteogenic induction was also assessed by determining alkaline phosphatase activity (D) and confirming the presence of a mineralized matrix by Alizarin Red staining (E) and hydroxyapatite staining (F). Images in D-F are representative of at least three independent biological repeats. Scale bar in F = 200µm.


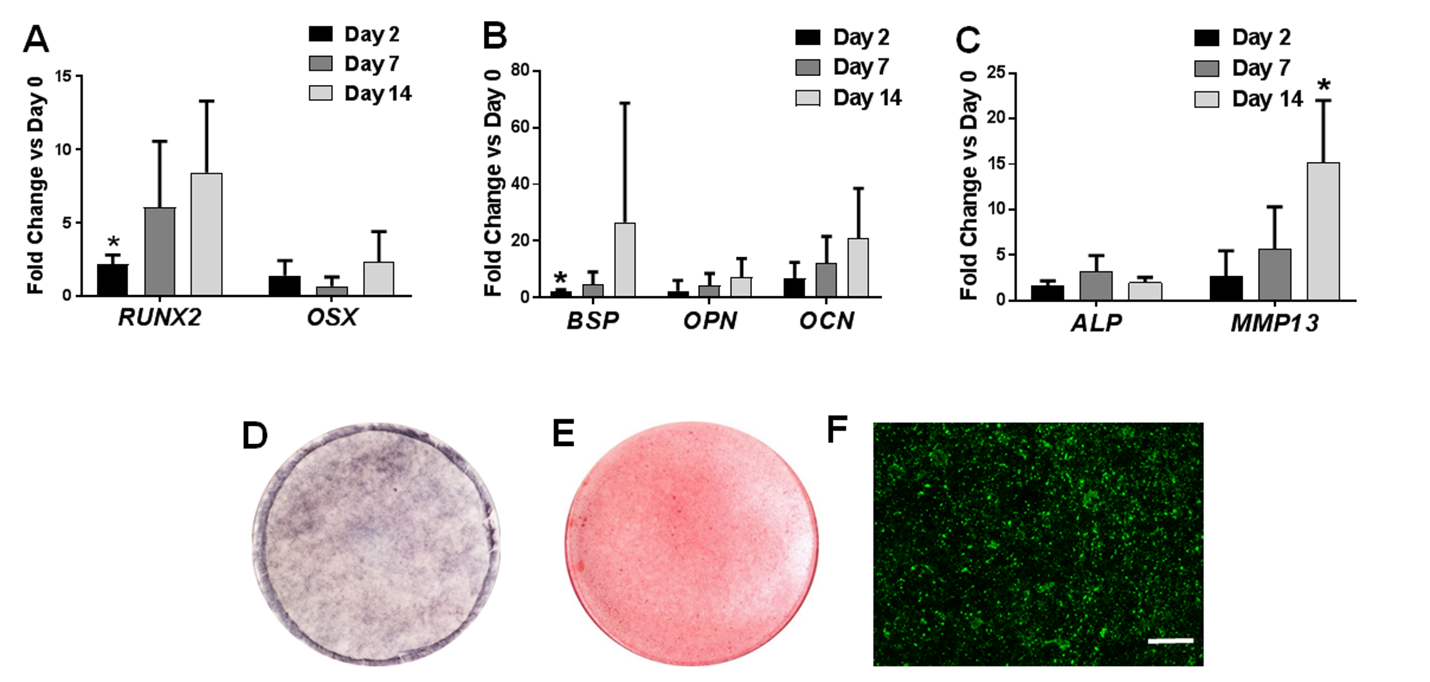


**Supplemental Fig. 3: Osteogenic induction of human dedifferentiated chondrocytes transduced with lentivirus expressing a non-silencing control RNA.** Expression of osteogenic genes (A-C) was assessed at day 2, 7, or 14 after osteogenic induction of DDCs. Each gene was normalized to *PPIA* and then calculated as fold change expression compared to day 0 (± SD; *n* = 3; * *p* < 0.05). Osteogenic induction was also assessed by determining alkaline phosphatase activity (D) and confirming the presence of a mineralized matrix by Alizarin Red staining (E) and hydroxyapatite staining (F). Images in D-F are representative of at least three independent biological repeats. Scale bar in F = 200µm.


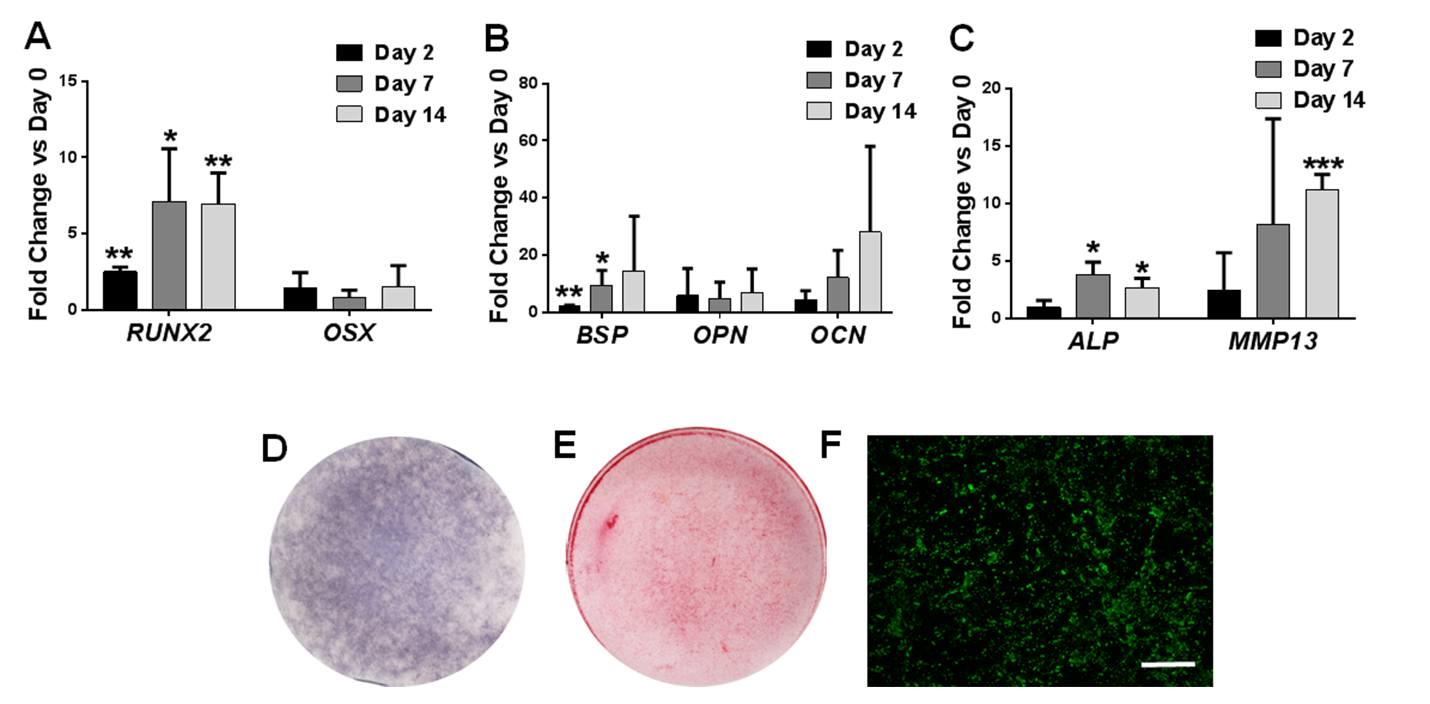


**Supplemental Fig. 4:** **Annotated KEGG graphs of significantly perturbed pathways.** KEGG graphs illustrating marked reductions in cell cycle (A) and regulation of actin cytoskeleton (B) as rendered by the R/Bioconductor package Pathview. Genes or complexes that are down-regulated versus background are rendered as blue and up-regulated genes and complexes are gold. The scale has been compressed to a maximum log 2 fold-change of 2 and minimum of -2 for readability.

**
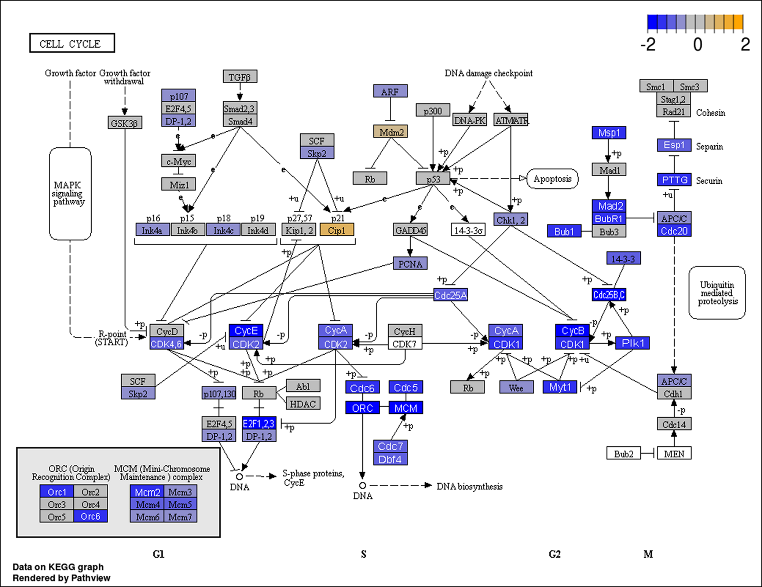
**

**
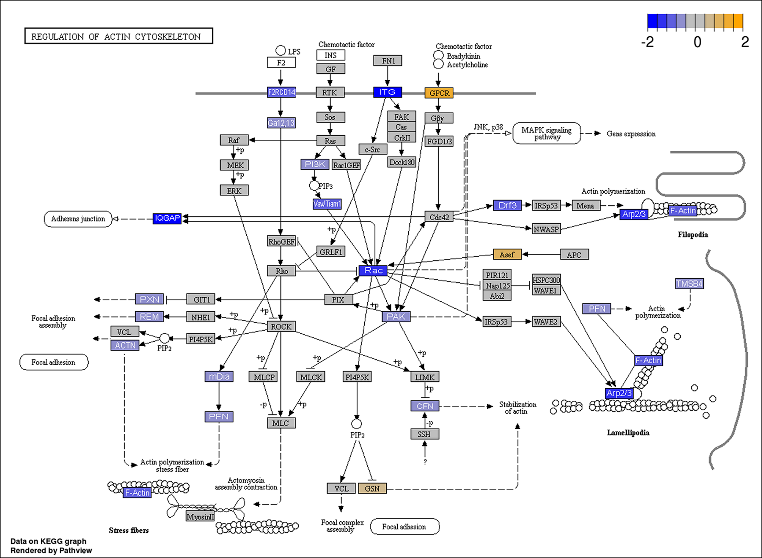
**

**Supplemental Fig. 5: Alizarin Red-stained scaffold tissue sections.** Representative light microscopy images showing differences in levels of calcified bone matrix on scaffolds generated by DDCs transduced with LV-NS, LV-138, or LV-138 + LV-RhoC. Paraffin sections of scaffolds were generated following 28 days in osteogenic induction media. Upper panel: 4x magnification; lower panel 20x magnification of the boxed area shown at 4x magnification.


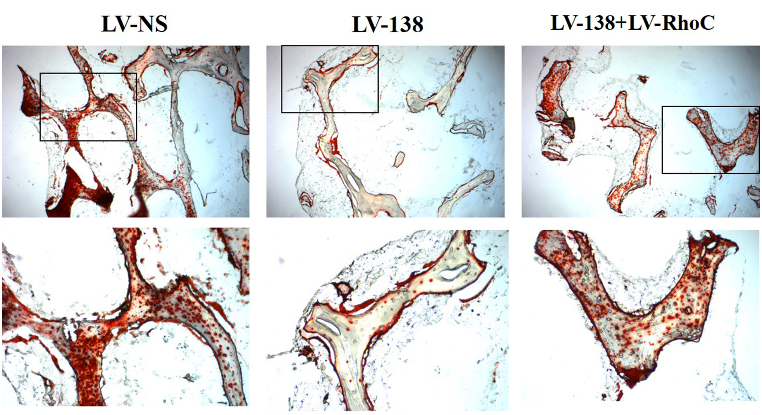

Supplement: Supplementary file 4 — Supporting Figures S1. [file JBM4-3-na-s004.docx]
